# Gene Set Enrichment Analysis (GSEA) of *Toxoplasma gondii* expression datasets links cell cycle progression and the bradyzoite developmental program

**DOI:** 10.1186/1471-2164-15-515

**Published:** 2014-06-24

**Authors:** Matthew McKnight Croken, Weigang Qiu, Michael W White, Kami Kim

**Affiliations:** Departments of Medicine, Microbiology & Immunology and Pathology, Albert Einstein College of Medicine, 1300 Morris Park Avenue, 10461 Bronx, NY USA; Department of Biological Sciences, Hunter College of the City of University of New York, New York, 10065 NY USA; Departments of Molecular Medicine and Global Health, University of South Florida, Tampa, 33612 FL USA

**Keywords:** Gene expression, Transcriptome, Parasite, Bradyzoite, Tachyzoite, Differentiation, Development

## Abstract

**Background:**

Large amounts of microarray expression data have been generated for the Apicomplexan parasite *Toxoplasma gondii* in an effort to identify genes critical for virulence or developmental transitions. However, researchers’ ability to analyze this data is limited by the large number of unannotated genes, including many that appear to be conserved hypothetical proteins restricted to Apicomplexa. Further, differential expression of individual genes is not always informative and often relies on investigators to draw big-picture inferences without the benefit of context. We hypothesized that customization of gene set enrichment analysis (GSEA) to *T. gondii* would enable us to rigorously test whether groups of genes serving a common biological function are co-regulated during the developmental transition to the latent bradyzoite form.

**Results:**

Using publicly available *T. gondii* expression microarray data, we created *Toxoplasma* gene sets related to bradyzoite differentiation, oocyst sporulation, and the cell cycle. We supplemented these with lists of genes derived from community annotation efforts that identified contents of the parasite-specific organelles, rhoptries, micronemes, dense granules, and the apicoplast. Finally, we created gene sets based on metabolic pathways annotated in the KEGG database and Gene Ontology terms associated with gene annotations available at http://www.toxodb.org. These gene sets were used to perform GSEA analysis using two sets of published *T. gondii* expression data that characterized *T. gondii* stress response and differentiation to the latent bradyzoite form.

**Conclusions:**

GSEA provides evidence that cell cycle regulation and bradyzoite differentiation are coupled. Δ*gcn5A* mutants unable to induce bradyzoite-associated genes in response to alkaline stress have different patterns of cell cycle and bradyzoite gene expression from stressed wild-type parasites. Extracellular tachyzoites resemble a transitional state that differs in gene expression from both replicating intracellular tachyzoites and in vitro bradyzoites by expressing genes that are enriched in bradyzoites as well as genes that are associated with the G1 phase of the cell cycle. The gene sets we have created are readily modified to reflect ongoing research and will aid researchers’ ability to use a knowledge-based approach to data analysis facilitating the development of new insights into the intricate biology of *Toxoplasma gondii*.

**Electronic supplementary material:**

The online version of this article (doi:10.1186/1471-2164-15-515) contains supplementary material, which is available to authorized users.

## Background

*Toxoplasma gondii* is an Apicomplexan parasite that is associated with encephalitis in the immunocompromised and chorioretinitis and birth defects in children exposed in utero. A central aspect of *T. gondii* virulence is its ability to persist as a latent slow-growing bradyzoite within tissue cysts. The reactivation of cysts, in the face of waning immune function, is a major cause of clinical toxoplasmosis. Despite the importance of this developmental transition the molecular mechanisms triggering differentiation are not understood. Expression analysis of bradyzoites and mutants unable to convert to bradyzoite have facilitated the identification of stage specific genes [1], but the critical signaling pathways have not yet been defined, in part because systems analysis tools are not available for this organism.

Gene expression analysis has revolutionized the analysis of biological problems, enabling an unbiased examination of gene expression on a genome-wide level. Initial analyses to detect biologically relevant but statistically robust changes in gene expression relied upon identification of changes in expression of single genes, usually using criteria that were designed to identify genes whose expression was altered most markedly and reproducibly. This resulted in lists of genes whose relation to each other was not obvious. As datasets expanded, methods to account for biological processes or genes whose expression were related in similar pathways or regulated by similar stimuli or perturbations were developed.

One of the most commonly used statistical methods is Gene Set Enrichment Analysis (GSEA) [2]. GSEA incorporates prior knowledge about biological states to create a priori gene sets that can be tested for concordant behavior in different biological conditions representing different phenotypes or different genotypes. Thus the co-regulation of genes that are functionally related, regulated by similar factors and conditions, or have another hypothesized biological link can be tested statistically.

The 8,814 genes of the *T. gondii* ME49 genome have been assigned Gene Ontology terms that assign gene products using a standard controlled vocabulary (http://www.geneontology.org) [3] that is meant to allow comparisons of gene attributes across species and databases. While GO terms are useful, many genes (48.6%) have been annotated only as hypothetical proteins and a substantial number of genes belong to Apicomplexan-specific gene families, making GO vocabulary less useful for deducing the functions of many Apicomplexan genes. Most gene annotation of *T. gondii* has been computational with incorporation of community input via user comments. Extensive manually curated annotations like those available to model organism communities such as the Saccharomyces Genome Database available to the yeast community (http://www.yeastgenome.org/) [4] have not been uniformly incorporated into GenBank entries.

To develop gene sets that collate the extensive resources of ToxoDB (http://www.toxodb.org) [5], the primary community database, and published literature, we developed gene sets for our gene expression analysis, using the Molecular Signatures Database (MSigDB) (http://www.broadinstitute.org/gsea/msigdb/index.jsp) [2] that has been developed for use with GSEA, as a model. Application of these gene sets to analysis of preexisting datasets, illustrates a strong link between bradyzoite regulation and cell cycle and provides additional insight into the hierarchy of genes that lead to developmental transitions in *T. gondii.*

## Methods

### Gene Set Enrichment Analysis (GSEA)

Gene Set Enrichment Analysis is supported by the Broad Institute website (http://www.broadinstitute.org/gsea/index.jsp) [2] and includes versions compatible with Java, R or Gene Pattern. All GSEA analyses presented here were performed using the Java GSEA implementation. No experiments involving animals, humans, or human material were performed and therefore no ethics approvals were required for this study.

### Development of gene sets

The concept underlying GSEA is that genes that are somehow functionally linked will respond coordinately to a biological manipulation, in a manner that can be statistically detected and correlated to biological phenotype. These gene lists can be made using user-defined criteria. To define each new gene set, we first identified characteristics of interest in *T. gondii* and then identified genes that have or are associated with this characteristic. The default parameters of GSEA using gene lists of 15 to 500 genes were used as our target size in generating the new gene sets. KEGG pathway genes for *T. gondii* were downloaded from the KEGG site (http://www.genome.jp/kegg-bin/show_organism?menu_type=pathway_maps&org=tgo) [6]. GO terms and genes were downloaded from http://www.toxodb.org. Additional information about *T. gondii* genes and gene lists were provided by Jeroen Saeij (MIT) who had independently culled additional gene set information from the proteomics and gene expression literature as well http://www.toxodb.org. In the case of KEGG pathways, the gene sets are primarily metabolic pathways although KEGG also has an annotated list named “Toxoplasmosis” that consists of genes from *T. gondii* linked to pathogenesis or host interaction, as well as genes for host pathways affected. Similarly, gene sets derived from GO terms are related either by genes’ identified biological process, cellular component, or molecular function.

Manually curated gene lists provided by the *Toxoplasma* research community and incorporated in into the community database http://www.toxodb.org were obtained from Omar Harb, University of Pennsylvania, and compared with lists generated by text searches of Toxodb using key words apicoplast, rhoptry, microneme, dense granule, ROP, RON, MIC, GRA [5, 7, 8]. The gene list obtained from http://www.ToxoDB.org using “apicoplast” as a key word was very large and it was unclear whether all were genuine apicoplast proteins, so a community annotation gene list was used instead. Organellar gene sets were organized by subcellular localization rather than biological processes (i.e. where they are, rather than what they are doing).

To generate gene sets associated with particular stages of the parasite life cycle, we examined gene expression data and grouped co-regulated genes into relevant gene sets. Two previous studies used microarray experiments to identify genes associated with bradyzoite [9] and sporozoite [10] developmental stages of the life cycle were used to develop “tachyzoite”, “bradyzoite” and “sporozoite/oocyst” gene lists. The strain used for these studies, the Type II M4 strain, was maintained in continuous cat to mouse to cat to maintain competence for all life cycle transitions, but as a consequence, in vitro populations maintained a background level of spontaneous differentiation and were not completely pure “tachyzoites” or “bradyzoites”. Type II strains are most frequently used to model the biology of the bradyzoite differentiation process. Duplicate biological replicates of mRNA were used by the authors to probe microarrays, and our initial gene sets relied on the authors’ statistical inferences. Genes that were significantly up-regulated under the same conditions were placed together into gene sets, while those that were down-regulated were placed into an opposing gene set (e.g. tachyzoites and bradyzoites). It should be noted that since the gene sets are user defined, the gene sets used for GSEA can be overlapping based upon user-defined criteria. To view the genes that overlap in our gene sets, a complete list of genes is provided in Additional file 1 as a spread sheet (Table S1 GeneMembership). Gene lists used for this analysis (Version 6 gene IDs) and for the current *T. gondii* genome release (Version 10) are also provided in Additional files 2 and 3.

### Test data

To test the usefulness of our newly developed *T. gondii* gene sets, we used published microarray data sets to identify pathway enrichments associated with the development from tachyzoites to bradyzoites in *T. gondii.* The user guide for GSEA recommends 7 replicates, which are generally not available, so we used datasets with at least 3–4 biological replicates. These included the data set GSE23174 [11], which compared RH (type I) in vitro bradyzoites to in vitro intracellular tachyzoites as well as extracellular tachyzoites. These RH strain parasites lack the *UPRT* gene and differentiate more readily to in vitro bradyzoites than their wild-type parent. Bradyzoite induction was induced by low CO_2_, high pH culture conditions (alkaline stress) [11].

We also tested data set GSE22100 for enrichment of our gene sets [12], which was used to support a study of GCN5A function. The authors characterized the transcriptome of a RH strain mutant lacking GCN5A (Δ*gcn5A*), one of two *T. gondii* GCN5 histone lysine acetyltransferases [12]. The authors showed that, while the wild-type parasites and Δ*gcn5A* mutants show virtually identical transcriptomes when grown in tachyzoite conditions, when grown in alkaline stress wild-type parasites up-regulate several key markers of bradyzoite differentiation while the Δ*gcn5A* mutants fail to upregulate bradyzoite markers [12].

Expression data for both sets of experiments was extracted and normalized from CEL files using the ExpressionFileCreator module from Gene Pattern a software platform available at the website of the Broad Institute (http://www.broadinstitute.org/cancer/software/genepattern/) [13]. We used RMA normalization.

## Results and discussion

### Cell cycle

The cell cycle of *Toxoplasma gondii* is known to be divergent from other model eukaryotes. It has a greatly abbreviated or absent G_2_ phase with DNA synthesis (S-phase) coupled directly to mitosis (M phase) [14]. Behnke and colleagues identified two sub-transcriptomes, G_1_ and S/M, based on a microarray study of synchronized parasites, sampling mRNA levels every hour for twelve hours [14]. The replication time of the RH strain of *T. gondii* is reported to be about eight hours [15]. Based on the microarray results from the twelve, one-hour time points, the authors fit a spline model to estimate transcript levels throughout the cell cycle in higher resolution [14]. From the twelve actual time points, the spline model extrapolates sixty “splined” time points, each representing a twelve minute increment. Using this spline model, we identified peak times of expression for cell cycle regulated genes.

**Figure 1:**
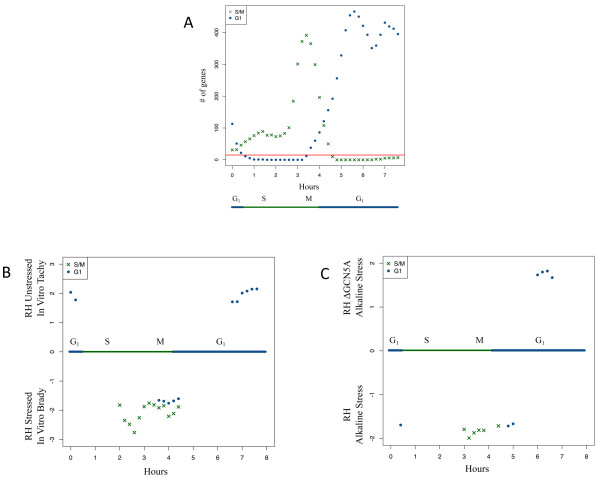
**Alkaline stress is associated with perturbation of cell cycle gene expression. A**: Cell Cycle Gene Set Sizes. Sizes of gene sets plotted against time of peak expression. Genes identified as S/M or G1 stage genes differentially expressed within the cell cycle [14] were assigned to gene sets that reflected genes expressed at different time points in the cell cycle. Assignments were made regardless of peak expression time, so that genes could belong to more than one gene set. Blue dots are G_1_ gene sets and green crosses indicate S/M gene sets. The red line indicates minimum size of gene set (15 genes) to be used with GSEA. Blue and green bars at bottom indicate phases of an eight hour RH strain tachyzoite cell cycle [14]. **B**: Cell Cycle Gene Expression of Tachyzoites and in vitro Bradyzoites. Gene expression arrays from tachyzoites and in vitro bradyzoites previously reported [11] were analyzed by GSEA. The plot shows normalized enrichment scores (NES) obtained from GSEA for those cell cycle gene sets with significant enrichment (FWER-adjusted p < 0.05) after GSEA analysis. A positive NES indicates that the gene set is associated with unstressed, tachyzoite parasites, while a negative NES indicates that the gene set is associated with alkaline stressed, in vitro bradyzoites. **C**: RHΔ*gcn5A* mutant cell cycle gene expression. Transcriptomes of alkaline stressed Δ*gcn5A* parasites and alkaline stressed wild-type tachyzoite [12] were compared by GSEA. Plotted normalized enrichment scores (NES) for those cell cycle gene sets with significant enrichment (FWER-adjusted p < 0.05). A positive NES indicates that the gene set is associated with RHΔ*gcn5A* mutant parasites lacking GCN5A; negative NES indicates that the gene set is enriched in the transcriptome of alkaline stressed parental wild-type RH parasites.

For each gene, we defined peak times of expression as greater than the mean expression across all splined time points plus 1.25 standard deviations. Genes assigned to each splined time point become our gene sets. Using these criteria, only 1,927 genes of the 2,833 cell cycle regulated genes are included in the gene sets. These criteria allowed us to generate gene sets for GSEA within the recommended size limits, between 15 and 500 genes. Figure 1A illustrates sizes of the generated gene sets for G_1_ and S/M and illustrates the previously reported major peaks of cell cycle gene expression [14]. A list of all genes and their membership in each GSEA gene set is in Additional file 1 and full gene sets for each cell cycle time point are available in Additional files 2 and 3 as gmt files compatible with GSEA.

We ran GSEA using the cell cycle gene sets against two different published microarray experiments. These datasets were chosen because the experiments were performed in different laboratories but used RNA extracted from the common laboratory strain RH and expression profiling was performed on the community generated *Toxoplasma* chip on the Affymetrix microarray platform [16]. These datasets illustrate cases where parasites modulate steady-state mRNA levels in response to environmental changes and test how genetically modified parasites respond to the same stimulus. We reasoned that our gene sets in combination with GSEA might provide additional insights into how transcriptional differences reflect biological responses to an environmental stress that is the standard laboratory manipulation for induction of in vitro bradyzoites.

In the first expression experiment reported by Lescault (GSE23174) [11], “wild-type” RH (Type I Δ*uprt*Δ*hxgprt*) parasites were grown in tissue culture under pH neutral, tachyzoite-conducive conditions (5% CO_2_) or under alkaline, bradyzoite-inducing conditions (low CO_2_) [11]. RH strain parasites lacking *UPRT* are more sensitive to alkaline stress induction of bradyzoites [17] whereas most laboratory strains of RH do not complete bradyzoite differentiation, although they can express classic bradyzoite markers such as BAG1 when grown under alkaline stress conditions for 3–4 days. Comparing intracellular tachyzoites to in vitro “bradyzoites,” gene sets associated with the S/M phase, are strongly up-regulated under alkaline stress “bradyzoite” conditions. These observations are consistent with the prolongation of the S/M phase associated with bradyzoite differentiation noted previously [18]. This prolonged S/M is distinguished by the coexpression of both tachyzoite (SAG1) and bradyzoite (BAG1) by the same parasite [18]. Asynchronous tachyzoite cultures are usually typically predominantly (about 70%) in G1 of the cell cycle. Notably, some gene sets classified as G_1_ whose temporal expression coincides with the S/M phase are up-regulated in the in vitro bradyzoites (Figure 1B).

We also tested for perturbation of cell cycle associated genes using data set GSE22100 that compared the transcriptomes of mutant parasites lacking GCN5A, a histone acetyltransferase, to wild type parasites (in this case RHΔ*hxgprt*) grown in alkaline stress [12]. GCN5A is required for activation of bradyzoite-specific genes and Δ*gcn5A* mutant parasites fail to express bradyzoite differentiation markers in alkaline culture and exhibit transcriptomes that are “tachyzoite-like” [12]. Using GSEA, we observe a significant enrichment of S/M gene sets within the wild-type, alkaline stressed parasites and G1 gene sets by the Δ*gcn5A* mutant parasites exposed to alkaline stress (Figure 1C), indicating that under alkaline stress, the expression of Δ*gcn5A* mutant cell-cycle regulated genes is more similar to tachyzoites. This may indicate that one of the mechanisms by which GCN5A regulates the stress response is via regulation of cell cycle checkpoints.

### Oocyst maturation

Following the sexual phase of the *Toxoplasma* life cycle, oocysts are released in the cat’s feces [10]. Sporulation of fecal oocysts leads to infective, environmentally hardy parasites that can contaminate human food and water supplies. These oocysts are highly infectious and, when ingested, can cause clinically symptomatic infection, usually chorioretinitis, in both immunocompetent and immunosuppressed individuals. Using expression microarrays, Fritz et al. profiled a type II (strain M4) oocyst transcriptome immediately and at days four and ten after oocyst release [10]. By microscopy, day zero oocysts are immature and completely unsporulated. Day four oocysts begin to develop a more mature structure, but less than half have sporozoites. Day ten oocyst are considered mature, in that almost all have two well developed sporocysts and the “vast majority” had discernible sporozoites [10]. Following the development of the oocyst, a serial transcriptomic study was performed to identify several different patterns of gene expression during oocyst development.

**Figure 2:**
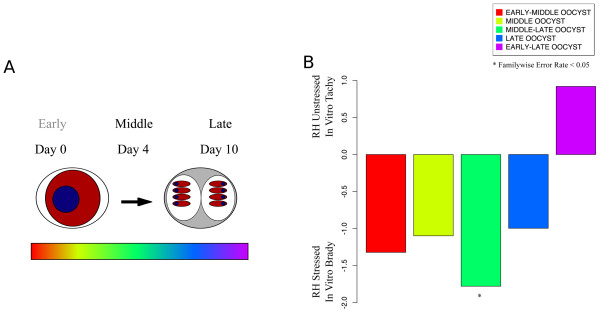
**Oocyst development induces genes in common with bradyzoite development. A**: Gene sets associated with oocyst sporulation. Fritz et al. assayed the transcriptome of oocysts at days 0, 4, and 10 after being expelled by the feline host [10]. We developed five gene sets based upon day(s) of peak expression as described in the materials and methods. The “early” gene set was excluded because too many genes (>500) fell into this group. Additional file 1 shows members of the gene sets and the patterns of expression of oocyst gene sets are shown in Additional file 4. The gradient of color indicates the approximate time of peak expression of the gene sets used (red: early-middle; light green: middle; green: middle-late: blue late; purple: early-late). **B**: The transcriptome of RH in vitro bradyzoites is enriched for genes associated with middle-stage oocysts. Plotted normalized enrichment scores (NES) for oocyst development gene sets for the datasets reported by Fritz [9]. A positive NES indicates that the gene set is associated with unstressed, tachyzoite parasites, while a negative NES indicates that the gene set is associated with alkaline stressed, in vitro bradyzoites as reported by Lescault [11]. Stars indicate significant enrichment (FWER-adjusted p < 0.05). The color of the bar corresponds to the indicated colors in panel A that depict the approximate time of peak expression of the gene sets used (red: early-middle; light green: middle; green: middle-late: blue late; purple: early-late).

We assigned genes to thirteen sporozoite gene sets based on changes in expression between time points as described in (Additional file 4: Figure S1). Five of these are “core” oocyst gene sets, featuring genes whose expression peaked during one or two of the observed time points (Figure 2A). These gene sets were called “early-middle”, “middle”, “middle-late”, “late”, and “early-late” oocyst genes. Too many genes fell into the “early” gene set to be useful for GSEA. An additional seven gene sets were created to encompass genes with more complex patterns of expression. These gene sets are provided as extended oocyst gene sets in Additional files 1, 2 and 3 and their corresponding patterns of expression are illustrated in Additional file 4.

**Figure 3:**
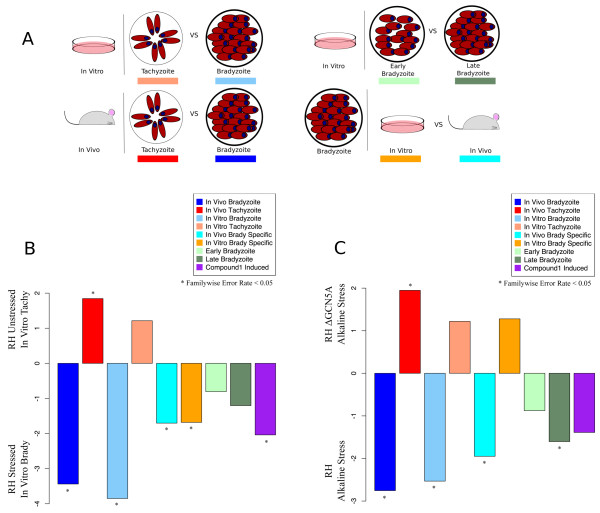
**Identification of patterns of bradyzoite associated gene expression. A**: Gene sets associated with tachyzoite-bradyzoite transition. Eight gene sets were derived from the microarray analysis encompassing gene sets with differential expression during the tachyzoite and bradyzoite stages [9]: Genes up-regulated in either 1) in vitro tachyzoites or 2) in vitro bradyzoites were each placed into their own gene sets. Genes up-regulated in either 3) in vitro tachyzoites or 4) mouse-derived bradyzoites were each placed in their own gene sets. Genes up-regulated in either 5) in vitro day 4 bradyzoites or 6) in vitro day 8 bradyzoites were each placed in their own gene sets. Genes up-regulated in either 7) in vitro bradyzoites or 8) mouse-derived bradyzoites were each placed in their own gene sets. Colored lines under each picture correspond to the color coding of enriched gene sets in Panels **B** and **C**. **B**: The transcriptome of alkaline stressed RH parasites is enriched for bradyzoite gene sets. Transcriptomes of wild-type RHΔ*uprt*Δ*hxgprt* tachyzoites and alkaline stressed RHΔ*uprt*Δ*hxgprt* parasites [11] were compared. GSEA-derived normalized enrichment scores (NES) for bradyzoite gene sets are shown. A positive NES indicates that the gene set is associated with unstressed, tachyzoite parasites, negative NES indicates that the gene set is associated with alkaline stressed, in vitro bradyzoites. Color coding of enriched gene sets is identical to that shown in Figure 3A. Stars indicate significant enrichment (FWER-adjusted p < 0.05). **C**: The transcriptome of RHΔ*gcn5A* parasites is enriched for tachyzoite gene sets under alkaline stress. Plotted normalized enrichment scores (NES) for bradyzoite gene sets. A positive NES indicates that the gene set is associated with Δ*gcn5A* mutant RH parasites missing the GCN5A enzyme [12], while negative NES indicates that the gene set is associated with the parental wild-type RH. Stars indicate significant enrichment (FWER-adjusted p < 0.05).

We ran GSEA with the previously described microarray experiments (GSE23174 and GSE22100) against the oocyst gene sets. Surprisingly, in the comparison of the Lescault “tachyzoites” with “bradyzoites” [11], genes associated with the middle-late stage of sporulation were enriched in the in vitro bradyzoite parasite population (Figure 3B). In contrast, GSEA analysis of the Δ*gcn5A* mutant parasites showed no significant enrichment for any oocyst gene set (data not shown). Too little is known about the sporozoite developmental program to definitively interpret the biological significance of these results, but one can hypothesize overlap between the in vitro bradyzoite and oocyst transcriptomes or that common genes are induced during any developmental transition. The Δ*gcn5A* RH background strain (RHΔ*hxgprt*) expresses stress markers associated with the bradyzoite transition, but does not complete differentiation to bradyzoites as defined by presence of the cyst wall [12].

### Bradyzoite differentiation

The ability of *Toxoplasma gondii* to differentiate into bradyzoites and persist within quiescent tissue cysts is a key survival strategy. The latent bradyzoite form allows the parasite to evade the host immune response while awaiting contact with a new host. Recrudescence of encysted parasites is responsible for most clinical disease and can cause lethal encephalitis in immunocompromised individuals. Therefore, the molecular mechanisms responsible for differentiation between tachyzoites and bradyzoites are of keen interest.

Prior work has suggested that bradyzoite differentiation and cell cycle are coupled, with the first detectable initiation of the differentiation program occurring in S/M phase, just prior to mitosis. As bradyzoites mature, their metabolism slows. Parasites induced to differentiate in vitro tend to have a slowing of the S/M phase and parasites with dual expression of the bradyzoite marker BAG1 and the tachyzoite marker SAG1 are more likely to be in S/M phase [18]. Induction of bradyzoite markers is observed when tachyzoites are incubated as extracellular tachyzoites for prolonged periods [19], suggesting that these parasites are able to sense alterations in their environment and induce bradyzoite gene expression. Based upon the cell cycle profile of extracellular parasites, this signal is likely to be sensed by parasites in the G1 phase of the cell cycle. Bradyzoite differentiation requires replication [1], but eventually, mature bradyzoites within in vivo tissue cysts complete mitosis and then exit the cell cycle, entering the G_0_ phase [18].

In addition to greatly slowed metabolism and replication, the other major feature of bradyzoites is cyst formation. During encystation within the host cell, the parasitophorous vacuole is lined with a glycosylated cyst wall, and the parasite expresses a stage-specific antigens, notably BAG1 (bradyzoite antigen 1) and CST1, a glycoprotein present in the bradyzoite cyst wall [20, 21]. Detection of these markers is considered diagnostic of bradyzoite differentiation. *T. gondii* also expresses paralogous metabolic genes associated with a particular stage of life. For instance, enolase 2 and lactose dehydrogenase 1 are expressed during tachyzoite development while enolase 1 and lactose dehydrogenase 2 are up-regulated following differentiation [1].

To obtain a more comprehensive view of bradyzoite differentiation, Buchholz and colleagues assayed the transcriptomes of type II (strain M4) bradyzoites [9]. They examined in vitro bradyzoites at four and eight days post induction, in vivo bradyzoites harvested from mouse brains twenty-one days post-infection, and compared the transcriptome of each bradyzoite type to the transcriptome of tissue culture grown tachyzoites [9]. This study catalogued changes in mRNA expression between tachyzoites and bradyzoites, differences between in vitro and in vivo derived bradyzoites, as well as temporal changes in the parasite transcriptome during bradyzoite development in tissue culture [9].

We created eight bradyzoite gene sets based on four different pair-wise comparisons illustrated in Figure 3A. These gene sets were designed to test whether bradyzoite differentiation had occurred, to identify which if genes associated with early or late stages of bradyzoite differentiation were expressed, as well as whether we could detect differences in expression of genes differentially expressed between mouse-derived cysts and alkaline/low CO_2_ in vitro cysts. As a final bradyzoite gene set, we used the set of genes enriched by treatment of parasite cultures with Compound 1, a kinase inhibitor that induces bradyzoite formation in Type II and Type III strains [22]. The Compound 1 gene set is based on previously published experiments examining differentiation in three different strains. Significance calls were made based on ANOVA analysis of all three strains [22], rather than pairwise comparisons like the other eight gene sets. We identified many common markers of bradyzoite differentiation within our bradyzoite gene sets. The complete gene sets can be found in Additional files 1, 2 and 3.

GSEA was able to clearly distinguish between tachyzoite and bradyzoite populations as characterized by Lescault (GSE23174) [11], but could not successfully identify these as in vitro bradyzoites and tachyzoites (Figure 3B). Further, the in vitro bradyzoite population was enriched for both in vitro and in vivo specific bradyzoite markers. This suggests that, although these genes are differentially expressed by in vitro and in vivo bradyzoites, both sets of genes are more highly expressed in bradyzoites than tachyzoites, regardless of the origin of the bradyzoite. The Compound 1 induced gene set was significantly enriched in the bradyzoite population.

GSEA classified the transcriptome of Δ*gcn5A* parasites as tachyzoite-like and that of the parental RH strain as bradyzoite-like (Figure 3C). This is consistent with Naguleswaran and colleague's observation that the mutant parasites fail to tolerate alkaline stress conditions [12]. Curiously, wild-type RH is enriched for in vivo specific bradyzoite markers, but not in vitro specific genes, suggesting subtle differences in mRNA expression profiles. It also interesting to note that in vitro specific bradyzoite gene set is enriched in the more tachyzoite-like Δ*gcn5A* parasites, albeit non-significantly (p = 0.259). The basis of this enrichment is unclear. The genes most up-regulated are diverse, but include DNA replication factors, protein translational machinery, and other apparently cell-cycle regulated genes.

### Subcellular localization

Prior studies in both *Plasmodium* [23, 24] and *T. gondii* [14] showed that steady state mRNA levels are present “just in time” with related metabolic genes or organellar genes frequently expressed at similar points in the cell cycle. We collected genes associated with the secretory organelles as prior microarray analysis of the gene products that localize to: rhoptries, micronemes, and dense granules were often coexpressed. In addition, we used a set of genes that was a part of the community annotation of gene products that localize to the apicoplast (obtained from Omar Harb, http://www.toxodb.org). Taken together, we have gene sets describing many of the cellular structures specific to Apicomplexans. These gene lists can be revised as experimental evidence accumulates about these organelles, and additional hypothetical genes are assigned to secretory organelles, the apicoplast, or the mitochondrion [25].

The in vitro bradyzoites of Lescault and colleagues (GSE23174) [11] show enrichment for gene products localized to micronemes and rhoptries, while their tachyzoite counterparts show up-regulation of denizens of the apicoplast (Additional file 5: Figure S2A). We did not observe any significant enrichment in either the wild-type or Δ*gcn5A* alkaline stressed parasite populations for any of the examined organellar gene sets although the trends of gene expression enrichment were evident (Additional file 5: Figure S2B).

### Metabolic pathways

Both the Gene Ontology (GO) and Kyoto Encyclopedia of Genes and Genomes (KEGG) projects have been applied to the *Toxoplasma* genome to better characterize the functions of and the relationships between genes. KEGG contains a database of metabolic pathways to which homologous genes from any species may be mapped onto a “canonical” pathway [6]. Although there are obvious drawbacks to this approach when dealing with a divergent eukaryote like *T. gondii*, it does provide a framework for examining parasite metabolism. Of the 87 annotated KEGG pathways present in *Toxoplasma*, only 33 have an appropriate size for GSEA (See Additional files 1, 2 and 3). Further manual annotation of metabolic pathways and comparison with other databases such as the LAMP database [26], will likely improve our knowledge of *Toxoplasma* metabolism. It should be noted that mis-assignment of one or two genes within these pathways would not necessarily affect the statistical ability of GSEA to detect co-regulation, if the majority of genes are correctly assigned and their steady state mRNA levels are co-regulated. GO terms are an effort to standardize descriptions of gene functions and place these functions into hierarchies, called ontologies [3]. This type of organization readily lends itself to GSEA. Of 325 GO terms assigned in *Toxoplasma*, 215 have an appropriate size for enrichment analysis (Additional file 1). For analysis of the datasets in this manuscript, GSEA using KEGG gene lists were more informative, but GO analysis is commonly used for inference of gene pathways in microarray analysis.

**Figure 4:**
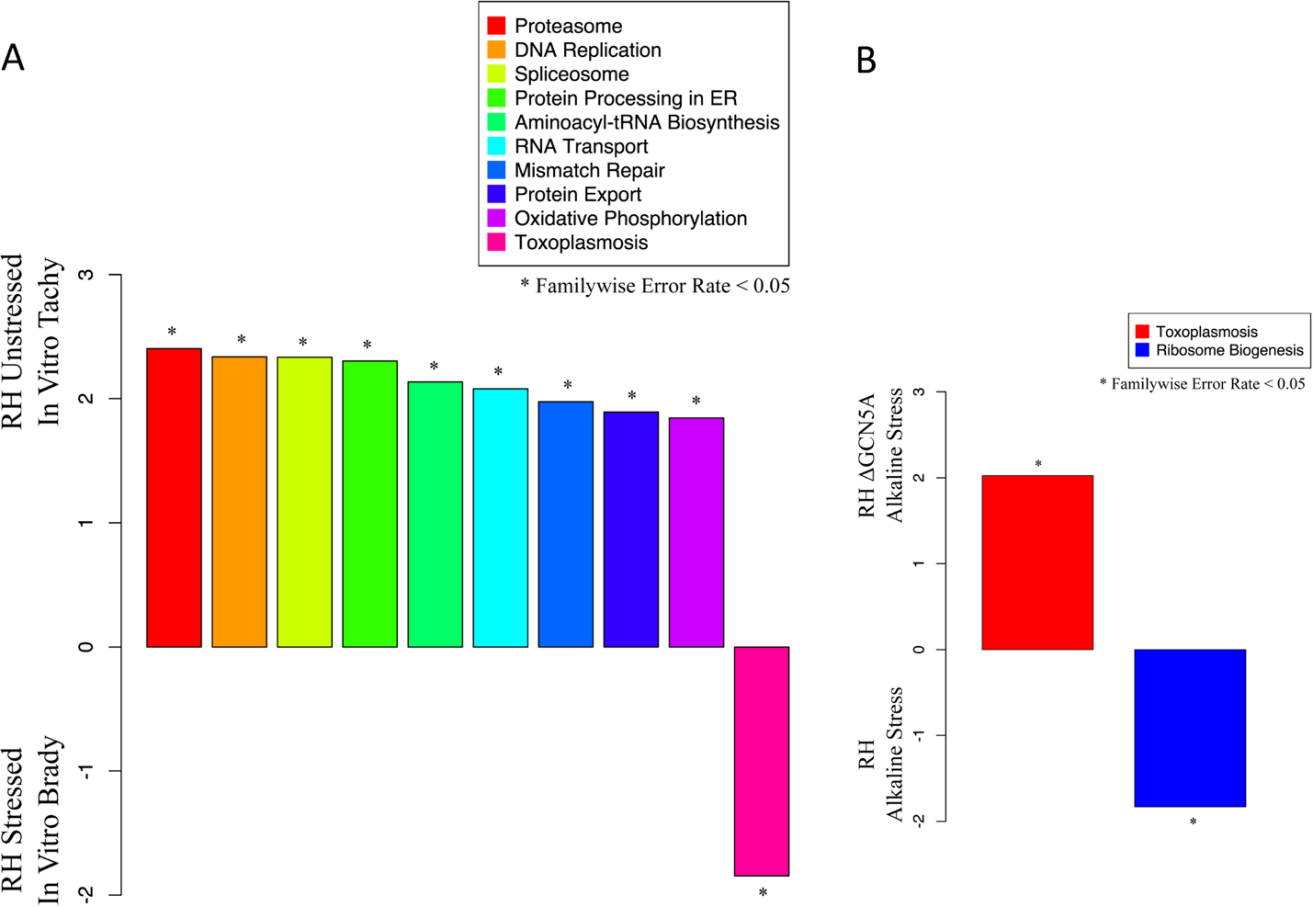
**Metabolic pathways affected by alkaline stress conditions. A**: KEGG pathways affected by bradyzoite induction. Plotted normalized enrichment scores (NES) for tachyzoite and bradyzoite gene sets after GSEA analysis of tachyzoites and in vitro bradyzoites as reported by Lescault [11]. A positive NES indicates that the gene set is associated with unstressed, tachyzoite parasites, while a negative NES indicates that the gene set is associated with stressed, in vitro bradyzoites. Stars indicate significant enrichment (FWER-adjusted p < 0.05). A number of metabolic pathway genes are differentially expressed. **B**: KEGG pathways affected by deletion of GCN5A. Plotted normalized enrichment scores (NES) for bradyzoite gene sets. A positive NES indicates that the gene set is associated with Δ*gcn5A* mutant RH parasites missing the GCN5A enzyme [12], negative NES indicates that the gene set is associated with the stress parental wild-type RH. Stars indicate significant enrichment (FWER-adjusted p < 0.05).

The tachyzoites tested by Lescault [11] (GSE23174) show significant enrichment of nine different KEGG pathways, while in vitro bradyzoites had enrichment of only one gene set (Figure 4A). This follows, since tachyzoites are highly metabolically active while bradyzoites are comparatively quiescent. These data may indicate key pathways that are affected during the transition to bradyzoites. We also see enrichment of transcripts associated with ATP production via oxidative phosphorylation and the citric acid cycle in tachyzoite parasites as well as transcription and translation machinery. Interfering with these pathways via drug, inducible mutation, or substrate starvation could induce bradyzoite differentiation. The generation of the atovoquone-resistant mutant R5, which differentiated more readily to bradyzoites, provides support for this hypothesis [27].

Similar experiments or reanalysis of previous experiments should provide new information about the particular pathways and molecular decision making that leads to life cycle development within *Toxoplasma*. It is also likely that important bradyzoite-specific metabolic pathways are not annotated, and manual curation of gene sets may facilitate further insights into the regulation of stage-specific metabolic pathways during different life cycle stages.

When alkaline stressed, the tachyzoite-like Δ*gcn5A* mutants (GSE22100) and their differentiated wild-type counterparts [12] were enriched for only one gene set derived from KEGG terms (Figure 4B). The only KEGG gene set enriched in stressed Δ*gcn5A* mutants was a pathway titled “Toxoplasmosis”. This pathway consists largely of cell surface adhesins (some specific to bradyzoites, others peculiar to tachyzoites) as well as many secreted factors that interfere with host cell signaling. The pathway also contains many host cell signaling factors, mainly related to the immune response. Since the “Toxoplasmosis” gene set includes several different classes of genes, its use with GSEA is not entirely appropriate. In this case, enrichment is based almost entirely on the up-regulation of stage-specific SRS domain-containing proteins.

In the alkaline stressed wild-type parasites, “Ribosome Biogenesis” genes were up-regulated in comparison to unstressed parasites. The differences in induction of metabolic pathway genes in these two datasets in response to alkaline stress may reflect the differences between the two background strains in stress response including possibly the induction of stress-induced translational control that has been implicated in bradyzoite cyst formation [28, 29].

### Extracellular tachyzoites

**Figure 5:**
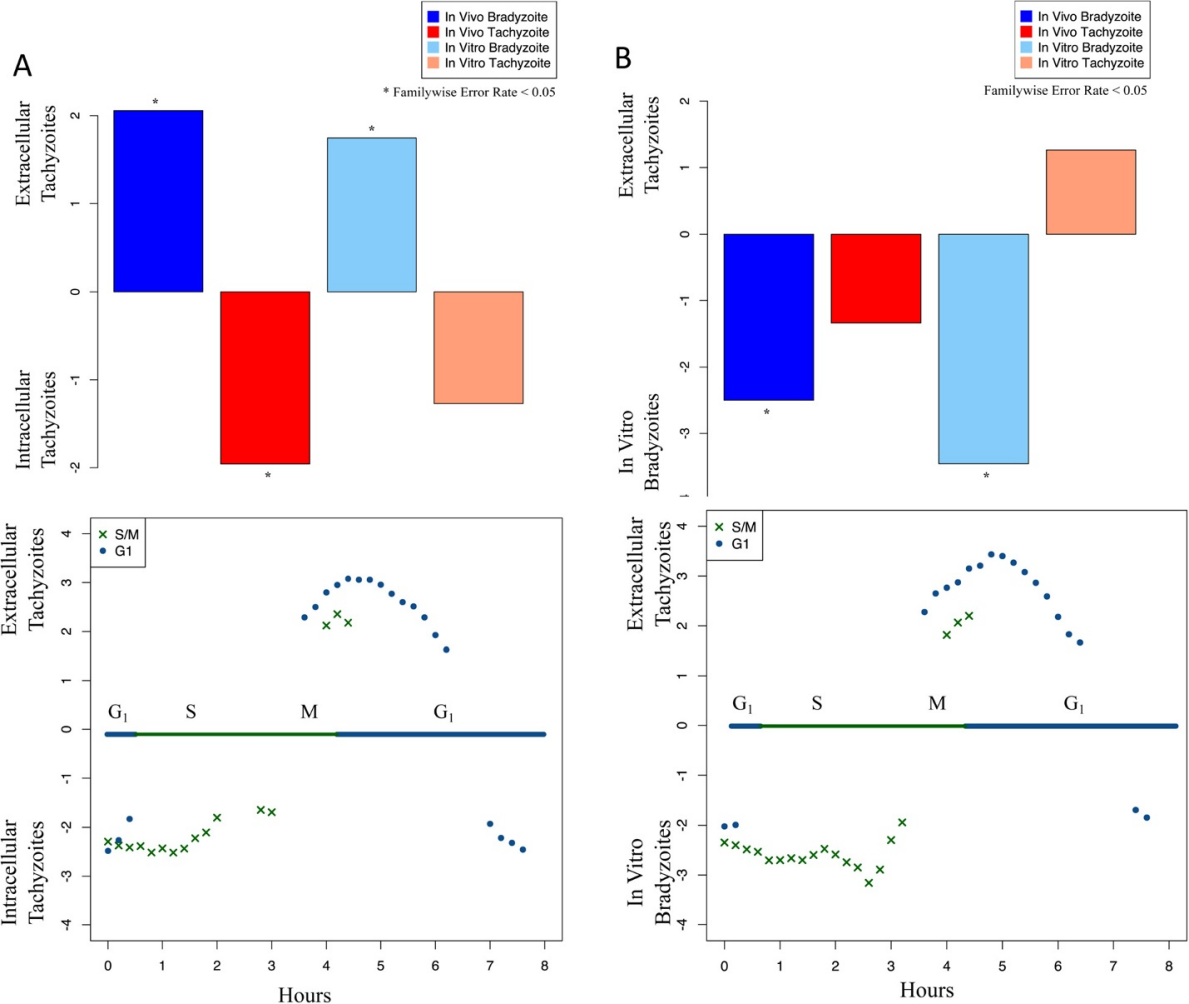
**Extracellular parasite gene expression profiles are distinct from those of intracellular parasites. A**: GSEA comparison of Extracellular and Intracellular Tachyzoites. Plotted normalized enrichment scores (NES) for tachyzoite/bradyzoite gene sets (top) and cell cycle gene sets (bottom). A positive NES indicates that the gene set is associated with extracellular tachyzoite parasites, negative NES indicates that the gene set is associated with intracellular tachyzoites. For tachyzoite/bradyzoite gene sets, stars indicate significant enrichment (FWER-adjusted p < 0.05). Cell cycle plots show only gene sets with significant enrichment. Relative to proliferating intracellular tachyzoites, extracellular tachyzoites are enriched in G1 genes and more “bradyzoite”-like. **B**: GSEA comparison of Extracellular Tachyzoites and in vitro Bradyzoites. Plotted normalized enrichment scores (NES) for tachyzoite/bradyzoite gene sets (top) and cell cycle gene sets (bottom). A positive NES indicates that the gene set is associated with extracellular tachyzoite parasites, negative NES indicates that the gene set is associated with in vitro bradyzoites. For tachyzoite/bradyzoite gene sets, NES are shown for gene sets with FDR <25% and stars indicate significant enrichment (FWER-adjusted p < 0.05). Cell cycle plots show only gene sets with significant enrichment. Relative to proliferating intracellular “bradyzoites”, extracellular tachyzoites are enriched in G1 genes and less “bradyzoite”-like.

Based on their mRNA expression experiments (GSE23174), Lescault and co-authors identified extracellular tachyzoites as a distinct stage of *Toxoplasma* asexual reproduction [11]. Using our newly developed gene sets, we further characterized the differences between extracellular tachyzoites, intracellular tachyzoites, and in vitro bradyzoites.When extracellular tachyzoites are compared to intracellular tachyzoites, bradyzoite gene sets, derived from in vitro and in vivo bradyzoite gene sets, are enriched in extracellular tachyzoites (Figure 5A). The in vivo tachyzoite gene set, but not the in vitro tachyzoite gene set, is enriched in the transcriptome of intracellular tachyzoites compared to extracellular parasites. However, when the transcriptome of extracellular tachyzoites is compared to the transcriptome of in vitro bradyzoites, bradyzoite gene sets are significantly enriched in the transcriptome of the bradyzoites, while tachyzoite gene sets are enriched in neither transcriptome (Figure 5B).

The transcriptome of extracellular tachyzoites also exhibit an apparent enrichment of G_1_ genes consistent with a post-mitotic cell cycle arrest or delay, when compared to both intracellular tachyzoites and intracellular in vitro bradyzoites. The timing of this arrest is consistent with cell-cycle exit in the G_0_ phase. Flow cytometry comparing DNA content of extracellular and intracellular tachyzoites shows a much greater proportion of haploid parasites (G_1_) in the extracellular group (Additional file 6: Figure S3). This confirms that extracellular parasites exist almost exclusively outside of the S phase and that their transcriptome is different from that of proliferating intracellular tachyzoites [30].

Taken together, these GSEA results support the hypothesis that extracellular tachyzoites represent a distinct state of parasite asexual development [11]. The GSEA results further suggest a continuum of stress response or stress preparedness between the three developmental stages examined in these experiments. At one end of the spectrum is the bradyzoite stage, which provides long-term defense against stress and host immunity, with replicating intracellular tachyzoites at the other end. The transcriptome of the extracellular tachyzoite stage is in the middle of this spectrum, reflecting the moderate stress encountered when the parasite is navigating the extracellular milieu to find a new host cell. Unlike the intracellular tachyzoite, these parasites lack the protection of a parasitophorous vacuole and host cell. However, unlike the bradyzoite stage, the extracellular stage is typically only short-term. Notably, sporozoites and bradyzoites, the other invasive zoite forms that must find new host cells also predominantly have G_1_ DNA content, a state that is likely to be the most advantageous for the parasite to successfully invade a new host cell without committing to proliferation. Cell cycle arrest may also be accompanied by upregulation of genes that enable the parasite to survive environmental stress prior to finding and invading a suitable host, with down-regulation of genes primarily required during intracellular replication.

## Conclusions

Deciphering the large amount of *Toxoplasma* transcriptomic data currently available requires integrative approaches to data analysis that incorporate experimental data and bioinformatics analysis. By establishing *T. gondii* gene sets, our aim is to create a framework to view changes in biological processes, rather than just individual genes, and drive hypothesis generation.

GSEA is a powerful tool for such knowledge-based analysis techniques and a considerable amount of relevant knowledge exists for *T. gondii*, but leveraging experimental data into gene sets can be a difficult task. The gene sets collected here describe a number of biological processes potentially linked to parasite virulence or disease pathogenesis. Because of our long-standing interest in the relationship of bradyzoite differentiation and cell cycle, we began have focused on biological aspects of these processes, particularly those specific to the parasite. Apicomplexan-specific or unique *T. gondii* genes may be associated with virulence or parasite metabolism, but differences in their expression are will be difficult to translate from gene sets developed for other model organisms. While KEGG and GO provide useful classifications for functionally related genes, assignment to KEGG pathways or GO terms are dependent on sequence homology between *T. gondii* and other eukaryotes. Given the evolutionary divergence of the Apicomplexa and the incomplete annotation of parasite genomes, new integrative approaches are needed that incorporate multiple lines and types of experimental data.

While these initial efforts have relied heavily on microarray expression data to develop *T. gondii* gene sets, expression analysis is by no means the only means for discovering functional links between genes. Protein-protein networks, inferred computationally or derived from experimental approaches such as proteomics analysis of immunoprecipitated complexes or yeast two-hybrid screens, may prove to be useful gene sets. A growing wealth of ChIP-chip and ChIP-seq data makes it possible to identify genes related by common epigenetic mark or bound by the same transcription factor. Finally, integration of knowledge from experiments validating existing gene sets can generate new groupings in an iterative manner. A major advantage of all of these strategies is that gene sets are user defined and therefore can evolve and be tested experimentally for statistical robustness.

The Molecular Signatures Database (MSigDB) curates many human gene sets for use with GSEA. MSigDB organizes the gene sets into seven separate collections. Many of these collections relate to oncology and immunology or other topics specific to metazoa that are not applicable to *Toxoplasma*, but others model gene sets that would be useful for *Toxoplasma*. In particular, genes that possess the same cis-regulatory elements may be grouped together as potential targets of the same transcription factor. Identification of cis-regulatory elements is ongoing within Apicomplexan parasites. A large family of candidate transcription factors, the ApiAP2, are conserved throughout the phylum Apicomplexa [31, 32]. The DNA binding specificities for most ApiAP2 in *Plasmodium falciparum* have already been described [33] and a similar effort for the *T. gondii* ApiAP2 binding specificities is nearly complete (Ranjan et al., in preparation). Simultaneous motif analysis and mapping of binding motifs onto the genome, should enable reconstruction of transcriptional regulatory pathways and produce sets of genes regulated by the same identical transcription factor. The gene sets described here represent a powerful new set of tools for deep analysis of *Toxoplasma* expression data with the GSEA software.

## Electronic supplementary material

Additional file 1: Table S1: GeneMembership. Gene sets used for GSEA. All *T. gondii* genes with their Version 6 ID are listed. The first column indicates classic tachyzoite (shaded blue) and bradyzoite markers (shaded yellow). Columns indicate the name of the gene set. (XLSX 204 KB)

Additional file 2: **GMT files formatted for use in GSEA (Version 10). Genome details were downloaded from the** ***T. gondii*** **genome resource** http://www.toxodb.org **.** The current genome Version 10 (R10) formatted Gene ID’s are provided. Detailed instructions for implementation of GSEA are available at the Broad Institute website (http://www.broadinstitute.org/gsea/index.jsp). (ZIP 66 KB)

Additional file 3: **GMT files formatted for use in GSEA (Version 6).** Genome details were downloaded from the *T. gondii* genome resource http://www.toxodb.org. Genome Version 6 (R6; used for this manuscript) formatted Gene ID’s are provided. Detailed instructions for implementation of GSEA are available at the Broad Institute website (http://www.broadinstitute.org/gsea/index.jsp). (ZIP 65 KB)

Additional file 4: Figure S1: Oocyst gene sets. Patterns of expression used to categorize core oocyst gene sets and extended oocyst gene sets. Expression data are from Fritz et al. [10] who profiled a type II (strain M4) oocyst transcriptome immediately and at days four and ten after oocyst release. (PDF 419 KB)

Additional file 5: Figure S2: Expression of organellar gene sets. Figure S2A: Bradyzoite induction leads to differential expression of organellar gene sets. Plotted normalized enrichment scores (NES) for bradyzoite gene sets from Lescault et al. [11]. A positive NES indicates that the gene set is associated with unstressed, tachyzoite parasites, while a negative NES indicates that the gene set is associated with alkaline-stressed in vitro bradyzoites. Stars indicate significant enrichment (FWER-adjusted p < 0.05). Figure S2B Alkaline-stressed RH Δ*gcn5A* parasites are not enriched for any subcellular gene sets. Plotted normalized enrichment scores (NES) for organellar gene sets. A positive NES indicates that the gene set is associated with alkaline-stressed RHΔ*gcn5A* parasites [12], while a negative NES indicates that the gene set is associated with the alkaline-stressed parental wild-type RH. None of the organellar gene sets tested had statistically significant enrichment (FWER-adjusted p < 0.05). (PDF 288 KB)

Additional file 6: Figure S3: Cell Cycle Profile (DNA Content) of Tachyzoites. Extracellular lysed tachyzoites or intracellular tachyzoites harvested from human foreskin fibroblasts were fixed and labeled with propidium iodide and analyzed by flow cytometry. The extracellular parasites are enriched for 1 N DNA content consistent with predominant G_1_ or G_0_ state. S phase parasites have intermediate amounts of DNA, whereas G_2_ or M parasites will have close to 2 N DNA content. Intracellular parasites are asynchronously proliferating with parasites in each of the major cell cycle stages, but are predominantly in G1. (PDF 742 KB)
