# Supplementary figures and images for: Gene Set Enrichment Analysis (GSEA) of Toxoplasma gondii expression datasets links cell cycle progression and the bradyzoite developmental program
Source: BMC Genomics. 2014 Jun 24;15(1):515. doi: 10.1186/1471-2164-15-515 (PMC4092224; doi:10.1186/1471-2164-15-515)

Figure S1

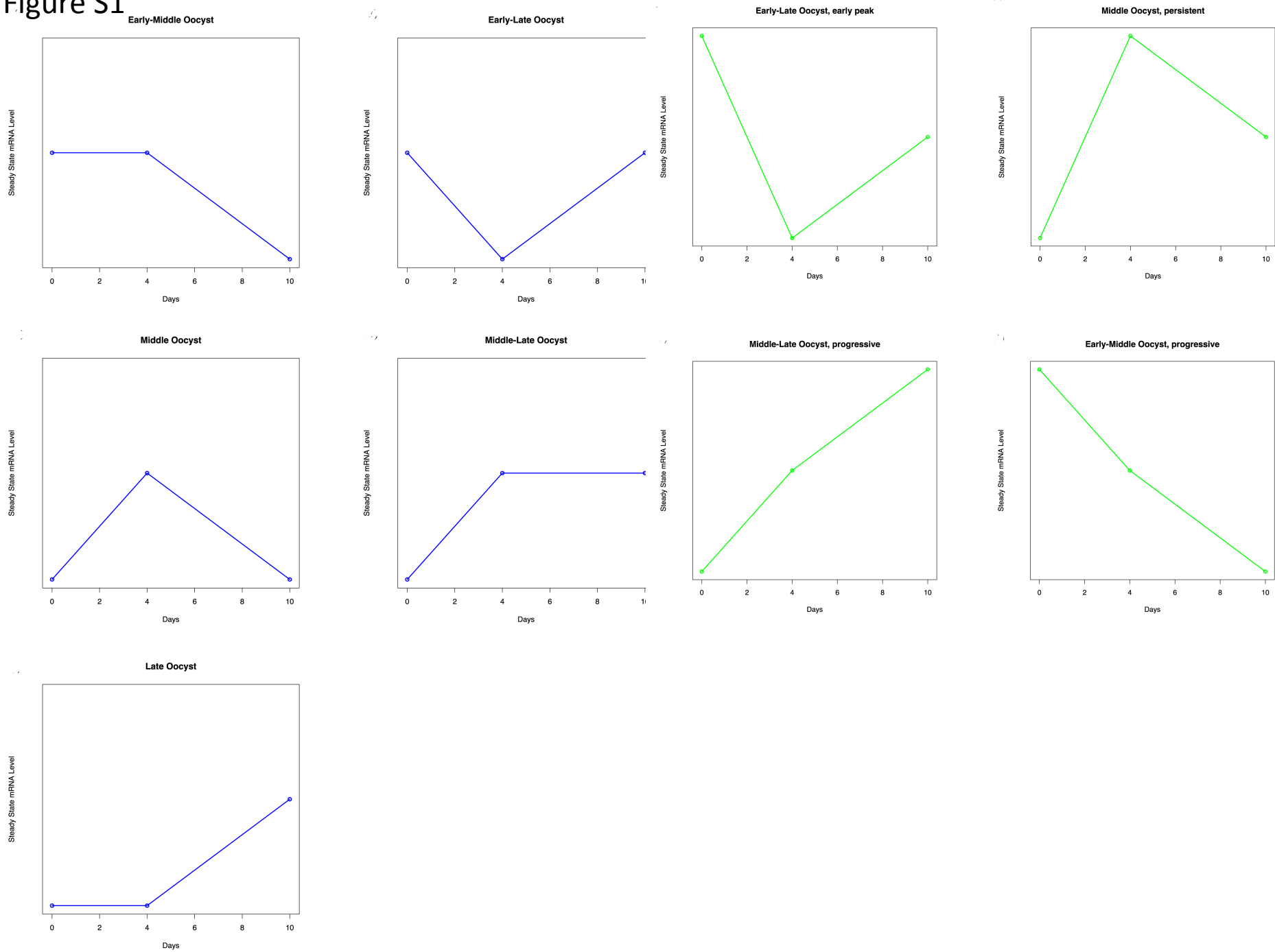

Supplement: Supplementary file 4 — Additional file 4: Figure S1: Oocyst gene sets. Patterns of expression used to categorize core oocyst gene sets and extended oocyst gene sets. Expression data are from Fritz et al. [10] who profiled a type II (strain M4) oocyst transcriptome immediately and at days four and ten after oocyst release. (PDF 419 KB) [file 12864_2014_6217_MOESM4_ESM.pdf]

Figure S2

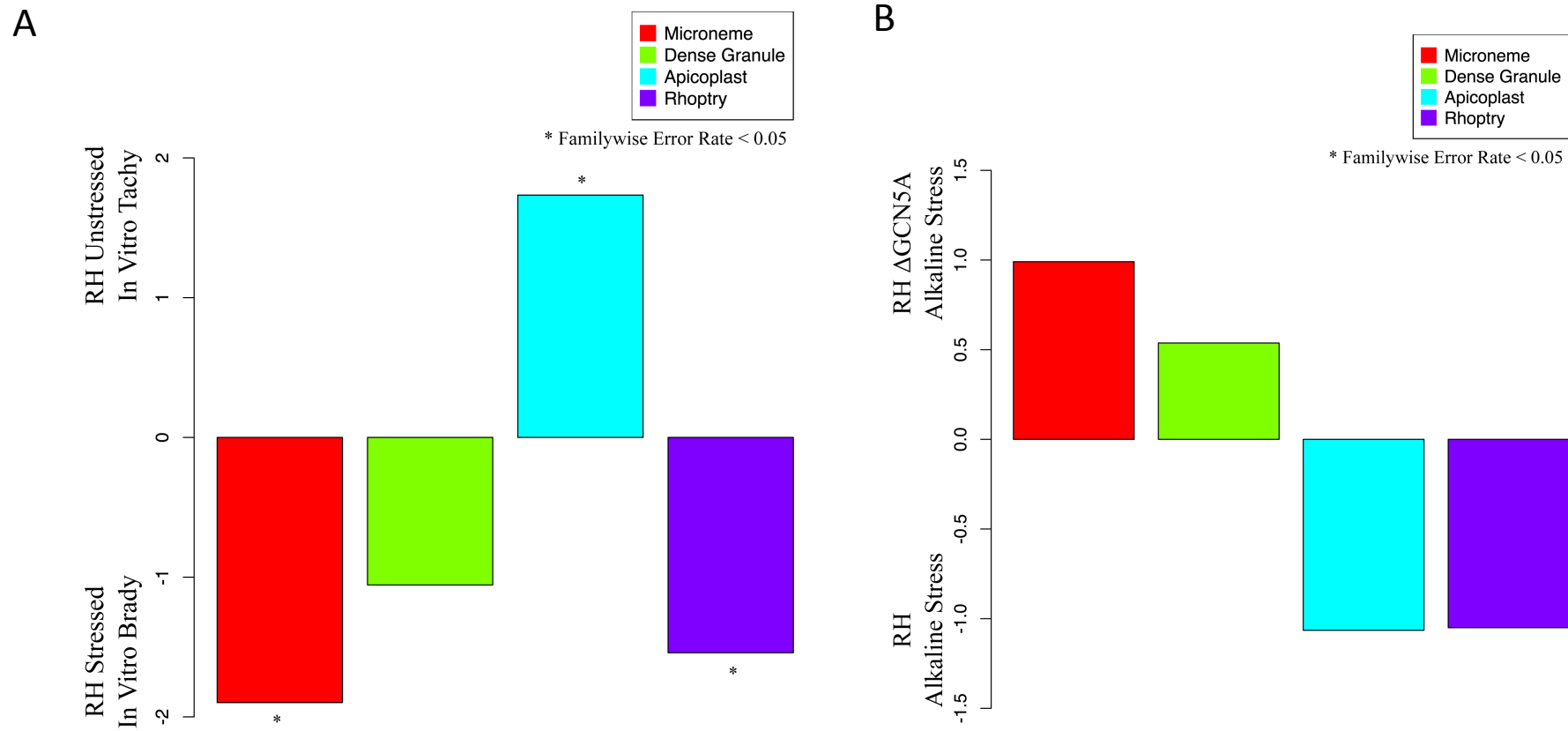

Supplement: Supplementary file 5 — Additional file 5: Figure S2: Expression of organellar gene sets. Figure S2A: Bradyzoite induction leads to differential expression of organellar gene sets. Plotted normalized enrichment scores (NES) for bradyzoite gene sets from Lescault et al. [11]. A positive NES indicates that the gene set is associated with unstressed, tachyzoite parasites, while a negative NES indicates that the gene set is associated with alkaline-stressed in vitro bradyzoites. Stars indicate significant enrichment (FWER-adjusted p < 0.05). Figure S2B Alkaline-stressed RH Δgcn5A parasites are not enriched for any subcellular gene sets. Plotted normalized enrichment scores (NES) for organellar gene sets. A positive NES indicates that the gene set is associated with alkaline-stressed RHΔgcn5A parasites [12], while a negative NES indicates that the gene set is associated with the alkaline-stressed parental wild-type RH. None of the organellar gene sets tested had statistically significant enrichment (FWER-adjusted p < 0.05). (PDF 288 KB) [file 12864_2014_6217_MOESM5_ESM.pdf]

Figure S3

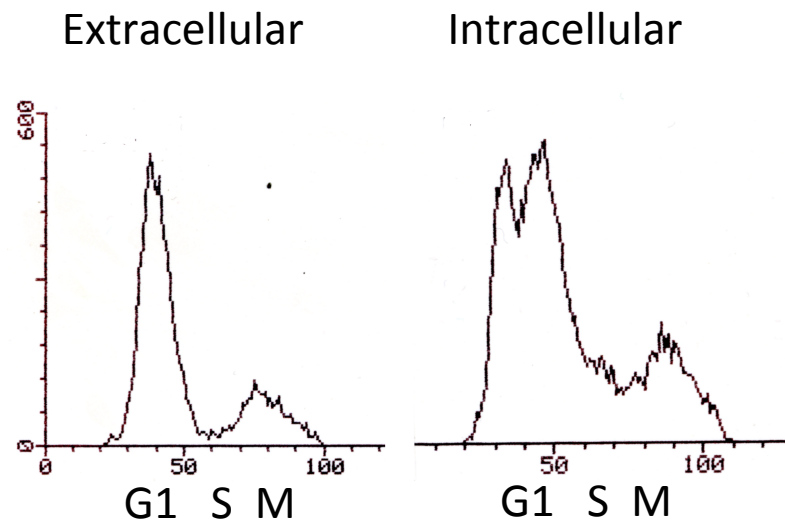

Supplement: Supplementary file 6 — Additional file 6: Figure S3: Cell Cycle Profile (DNA Content) of Tachyzoites. Extracellular lysed tachyzoites or intracellular tachyzoites harvested from human foreskin fibroblasts were fixed and labeled with propidium iodide and analyzed by flow cytometry. The extracellular parasites are enriched for 1 N DNA content consistent with predominant G1 or G0 state. S phase parasites have intermediate amounts of DNA, whereas G2 or M parasites will have close to 2 N DNA content. Intracellular parasites are asynchronously proliferating with parasites in each of the major cell cycle stages, but are predominantly in G1. (PDF 742 KB) [file 12864_2014_6217_MOESM6_ESM.pdf]
